# Salivary Antioxidants and Oxidative Stress in Psoriatic Patients: Can Salivary Total Oxidant Status and Oxidative Status Index Be a Plaque Psoriasis Biomarker?

**DOI:** 10.1155/2020/9086024

**Published:** 2020-01-03

**Authors:** Anna Skutnik-Radziszewska, Mateusz Maciejczyk, Katarzyna Fejfer, Julita Krahel, Iwona Flisiak, Urszula Kołodziej, Anna Zalewska

**Affiliations:** ^1^Doctoral Studies, Medical University of Bialystok, Poland; ^2^Department of Hygiene, Epidemiology and Ergonomics, Medical University of Bialystok, Poland; ^3^Department of Conservative Dentistry, Medical University of Bialystok, Poland; ^4^Department of Dermatology and Venereology, Medical University of Bialystok, Poland; ^5^Experimental Dentistry Laboratory, Medical University of Bialystok, Poland

## Abstract

The aim of our research was to evaluate redox balance parameters and biomarkers of oxidative stress (OS) in nonstimulated and stimulated saliva as well as the blood of patients with plaque psoriasis compared to healthy controls. The study involved 40 patients with plaque psoriasis and 40 generally healthy subjects matched by age and gender to the study group patients. We assayed the concentration/activity of antioxidant enzymes: salivary peroxidase (Px), catalase (CAT), and superoxide dismutase (SOD) measured in unstimulated saliva (NWS), stimulated saliva (SWS), and erythrocytes. In plasma as well as NWS and SWS, we measured the concentration/activity of reduced glutathione (GSH), total antioxidant potential (TAC), total oxidative status (TOS), oxidative stress index (OSI), and markers of oxidative modification of proteins: advanced glycation end products (AGE), advanced oxidation protein products (AOPP), and lipid oxidation products: malondialdehyde (MDA) and total lipid hydroperoxide (LOOH). In NWS and SWS, we also evaluated the rate of reactive oxygen species (ROS) production. The concentration of Px, CAT, and SOD was significantly higher in NWS of patients with plaque psoriasis vs. healthy subjects. In SWS of psoriatic patients, we observed considerably higher concentration of Px and CAT, and in erythrocytes of patients with plaque psoriasis, the concentration of GPx and CAT was significantly higher compared to that in the controls. The levels of AOPP, AGE, MDA, and LOOH were considerably higher in NWS, SWS, and plasma of the study group compared to the controls. The concentration of total protein and salivary amylase was significantly lower in NWS and SWS of psoriatic patients compared to the healthy control. In the course of plaque psoriasis, we observed redox imbalances with prevalence of oxidation reactions. Mechanisms involved in the synthesis/secretion of proteins and activity of amylase were depressed in both glands of psoriatic patients; however, they were more inhibited in the parotid gland compared to the submandibular gland. TOS concentration and OSI value in NWS and SWS may serve as diagnostic biomarkers of plaque psoriasis.

## 1. Introduction

Plaque psoriasis is a chronic recurrent systemic disease affecting 2–4% of the global population, in which inflammatory processes occur in the skin and are marked by the presence of abnormal skin patches that can significantly reduce the quality of life [1–3]. The pathophysiology of plaque psoriasis is of a complex and dynamic nature, and its key role is played by immunological and genetic factors, acute bacterial and viral infections, and chronic inflammation [4].

It is highlighted that one of the pathogenetic factors of psoriasis is also OS [5–7]. OS is a state of redox imbalance leading to oxidative damage to cellular components (such as proteins, lipids, and nucleic acids), which may entail cellular metabolism disorders and induce cell death by apoptosis [8]. OS is caused by both increased production of reactive oxygen species (ROS) and decreased concentration/activity of antioxidants responsible for their neutralization [9]. Free radical generation, disturbances of antioxidant barrier, and lipid peroxidation have been reported in the typical picture of plaque psoriasis. The early phase of active plaque psoriasis is characterized by intradermal infiltrates of activated polymorphonuclear leucocytes, followed by increased production of superoxide anion and other ROS [10]. The available evidence has also shown a decreased level of antioxidants, such as total blood thiols and serum vitamin E as well as antioxidant enzymes, such as serum, plasma and erythrocyte superoxide dismutase (SOD), plasma and erythrocyte catalase (CAT), and erythrocyte glutathione peroxidase (GPx) [6, 11–14]. Finally, the results of several studies revealed decreased total antioxidant capacity (TAC) in psoriatic patients compared to the controls [15, 16]. Some studies demonstrated an increased level of oxidative stress markers including plasma/serum lipid hydroperoxides, protein carbonyl, and nitric oxide end products [6, 15, 17]. High levels of plasma and red blood cell malondialdehyde (MDA) resulting from decreased activity of CAT and GPx were reported as markers of plaque psoriasis exacerbation [10, 18].

Plaque psoriasis is accompanied by pathological changes in the oral homeostasis, increasing the risk of periodontitis. It is also worth mentioning that plaque psoriasis has been linked to the dysfunction of salivary glands as well as changes in the saliva composition [19]. The pathogenesis of this disorder is not fully understood. However, taking into account the role of saliva in the oral cavity (initial food digestion, cleaning mucous membranes and teeth, maintenance of proper pH in the mouth, and participation in specific and nonspecific immunological and antioxidative defence processes), it seems advisable to recognize the pathomechanisms leading to disturbances in the function of salivary glands in the course of plaque psoriasis.

According to several studies, numerous biomarkers were considered for diagnosing plaque psoriasis; however, none of them proved valid and acceptable [15, 20]. Blood elements filtrated into the saliva have attracted much interest in this respect. The study of salivary parameters, in addition to blood, may also reflect a global picture of the body [21]. Moreover, the collection of saliva as a diagnostic material has numerous advantages over peripheral blood, the most important being noninvasive collection, lower anxiety of patients, and greater willingness to monitor one's health [9, 21, 22]. We demonstrated the usefulness of salivary redox balance biomarkers in the context of several general diseases (systemic sclerosis, insulin resistance, adulthood obesity, diabetes, dementia, and chronic kidney disease); however, there have been no studies to evaluate salivary redox markers and their usefulness in diagnosing psoriasis patients [9, 22–25].

The aim of our research was to evaluate redox balance parameters in nonstimulated and stimulated saliva as well as blood of plaque psoriasis patients compared to the healthy controls.

## 2. Materials and Methods

### 2.1. Patients

The study was approved by the Bioethics Committee of the Medical University of Bialystok, Poland (permission number: R-I-002/563/2018). All patients consented in writing to participate in the experiment.

40 patients with plaque psoriasis (27 men and 13 women), attending the Department of Dermatology and Venereology of the Medical University of Bialystok due to exacerbation of psoriatic symptoms, were qualified for the study. Within two years preceding the experiment, none of the patients had undergone any systemic antipsoriatic treatment, and they only had had topical treatment applied (keratolytic preparations, tar, local glucocorticosteroids, and vitamin D_3_ analogues). The extent and severity of skin lesions was assessed using the psoriasis area and severity index (PASI) that is usually implemented to measure the condition of skin changes.

The control group consisted of generally healthy people (*n* = 40, 27 men and 13 women) matched according to age, sex, and BMI to the study group patients. This group consisted of subjects reporting to the Specialist Dental Clinic of the Medical University of Bialystok for follow-up visits.

Patients with periodontitis (sulcus bleeding index (SBI) > 0.5, gingival index (GI) > 0.5) and pathological changes in the oral cavity mucosa (lichen-like, proliferative changes, skin erosion, and aphthous ulcers) were excluded from the study. Negative general medical history was a necessary factor to qualify for the experiment. The questionnaire completed by the patients included infectious diseases (hepatitis A, B, or C, HIV/AIDS), autoimmune diseases (Crohn's disease, Hashimoto's disease, and type 1 diabetes), and metabolic diseases (type 2 diabetes, gout, and osteoporosis) as well as diseases of the respiratory, cardiovascular, digestive, genitourinary, and coagulation systems.

In the six-month period preceding the study, patients and the healthy controls had not taken orally any nonsteroidal anti-inflammatory drugs, glucocorticosteroids, vitamins, other supplements, or antibiotics.

### 2.2. Diagnostic Material

The diagnostic material consisted of total, NWS, and SWS as well as the blood of patients from the control and the study group.

#### 2.2.1. Saliva Collection

On the day preceding the examination, patients were informed about the necessity to refrain from taking any medications for 6 hours before saliva collection. For 2 hours prior to the examination, patients had not consumed any food or drink other than water and had not performed any oral hygiene procedures. Saliva was collected via the spitting method in strictly defined and unchanging conditions: on the first day after admission to hospital, between 8 a.m. and 10 a.m., after at least a 5-minute adaptation period, in the same doctor's office, in the presence of the same dentists (A.S-R., K.F.), and in a sitting position with the head slightly inclined downwards. After rinsing the mouth twice with distilled water at room temperature, saliva was spat out into a test tube placed in ice. The duration of NWS collection was 10 minutes, and SWS 5 minutes. The saliva collected within the first minute was discarded [26]. Stimulation of saliva secretion was performed by sprinkling 10 *μ*L of 2% citric acid on the tip of the tongue every 30 seconds. Immediately after saliva collection, its volume was measured in a calibrated pipette with an accuracy of 100 *μ*L and then centrifuged (20 minutes, 3000 × g, +4°C; MPW 351, MPW Med. Instruments, Warsaw, Poland). To protect the samples against oxidation during their processing and storage, butylated hydroxytoluene (BHT, Sigma-Aldrich, Saint Louis, MO, USA; 10 *μ*L 0.5 M BHT/1 mL of saliva) was added to the obtained supernatants [22, 27]. Saliva samples for biochemical tests were frozen at -82°C and stored under these conditions until assayed (but not longer than six months). Moreover, the flow of NWS and SWS per minute was calculated by dividing saliva volume by the time of its collection.

#### 2.2.2. Blood Collection

Blood was collected on an empty stomach on the same day as, but prior to, the patient's examination and saliva collection. 7.5 mL of venous blood was collected in a S-Monovette® EDTA K3 tube (Sarstedt, Nümbrecht, Germany). Under laboratory conditions, the samples were centrifuged in order to separate the plasma from erythrocytes (1500 × g; 4°C, 10 minutes). Erythrocytes were rinsed three times with 0.9% cold NaCl (*v*/*v*) and then haemolysed by adding 50 mM cold phosphate buffer (pH 7.4) 1 : 9 (*v*/*v*) [28]. To prevent sample oxidation, 0.5 M BHT (Sigma-Aldrich, Saint Louis, MO, USA; 10 *μ*L/mL blood) was added to the blood [28]. The samples with signs of undesired haemolysis were excluded from the study. All samples were stored at -82°C (but not longer than six months).

### 2.3. Dental Examination

Dental examination was performed by the same dentists (A.S-R., K.F.) immediately after saliva collection, using a mirror, an explorer, and a periodontal probe (WHO, 621) in accordance with WHO criteria. The examination included dental status, DMFT (caries severity index) measurement of the total number of teeth with caries (D), teeth removed due to caries (M), and filled teeth (F). Additionally, we calculated PBI (papilla bleeding index) and GI according to Silness and Loe to determine the condition of the marginal periodontium based on the occurrence of bleeding as well as qualitative changes in the gums [29]. The interrater reliability for DMFT was *r* = 0.97, for PBI *r* = 0.96, and for GI *r* = 0.92.

### 2.4. Redox Analysis

The activity of antioxidant enzymes (Px, CAT, and SOD) was measured in NWS, SWS, and erythrocytes. Moreover, in the plasma, NWS, and SWS, we assayed TAC, total oxidant status (TOS), and oxidative stress index (OSI) as well as the concentrations of the markers of oxidative protein modifications: advanced glycation end products (AGE), advanced oxidation protein products (AOPP), and markers of oxidative lipid modifications: MDA and total lipid hydroperoxides (LOOH). In NWS and SWS, we additionally determined the rate of ROS production.

Unless stated otherwise, all analyses were performed in duplicate samples. Absorbance/fluorescence was measured with an Infinite M200 PRO Multimode Tecan microplate reader. All results were standardized by converting them to mg of total protein. Total protein concentration was determined using the bicinchoninic acid (BCA) method. Bovine serum albumin (BSA) was used as a standard (Pierce BCA Protein Assay; Thermo Scientific (Rockford, IL, USA)).

### 2.5. Antioxidant Barrier

Px (EC 1.11.1.7) activity was determined colorimetrically according to the method by Mansson-Rahemtulla et al. [30] based on the reduction of 5,5′-dithiobis-(2-nitrobenzoic acid) (DTNB) to thionitrobenzoic acid. The absorbance was measured five times at 30-second intervals at 412 nm.

The activity of GPx (EC 1.11.1.9) was determined spectrophotometrically based on the reduction of organic peroxides in the presence of NADPH [31]. It was assumed that one GPx unit decomposes 1 mol of NADPH per minute. The assay was performed at a 340 nm wavelength.

CAT (EC 1.11.1.6) activity was determined colorimetrically based on the rate of degradation of hydrogen peroxide (H_2_O_2_) in the sample [32]. One unit of CAT activity was defined as the amount of the enzyme that decomposes 1 mmol H_2_O_2_ per minute. The assay was carried out in triplicate tests at a 240 nm wavelength.

SOD (EC 1.15.1.1) activity was determined spectrophotometrically based on the rate of adrenaline oxidation to adrenochrome [33]. It was assumed that one SOD unit inhibits adrenaline oxidation by half. The determination was performed at a 480 nm wavelength.

GSH concentration was measured colorimetrically based on the reaction with DTNB [34]. The absorbance was read at a 412 nm wavelength.

### 2.6. Total Antioxidant/Oxidant Status and ROS Production

The level of TAC was determined spectrophotometrically using the radical cation of 2,2′-azino-bis(3-ethylbenzothiazoline-6-sulphonic acid) (ABTS) [35]. A calibration curve was prepared for 6-hydroxy-2,5,7,8-tetramethylchroman-2-carboxylic acid (Trolox). The assay was performed in triplicate tests at a 660 nm wavelength.

TOS level was determined bichromatically based on the oxidation of Fe^2+^ to Fe^3+^ in a sample [36]. The results were presented as micromole H_2_O_2_ equivalent per mg protein.

The OSI was calculated using the following formula: OSI = (TOS/TAC) × 100 [37].

The rate of ROS production was determined via a chemiluminescence test using luminol as an electron acceptor. Chemiluminescence was measured in 96-well microplates with black bottoms; H_2_O_2_ was used as a positive control [38].

### 2.7. Markers of Oxidative Protein Modification

AGE concentration was determined spectrofluorimetrically by measuring AGE-specific fluorescence at a 350/440 nm wavelength [39].

The concentration of AOPP was determined colorimetrically by measuring the oxidative capacity of iodine ions at a 340 nm wavelength [39]. For measuring AGE and AOPP concentrations, samples of NWS, SWS, and plasma were diluted in phosphate-buffered saline (PBS, pH 7.2) in a ratio of 1 : 5 (*v*/*v*).

### 2.8. Markers of Oxidative Lipid Modification

MDA concentration was determined colorimetrically with the TBARS method using thiobarbituric acid [40]. 1,1,3,3-Tetraethoxypropane was used as the standard. The determination was performed at a 535 nm wavelength.

The concentration of LOOH was determined spectrophotometrically with the FOX-2 test using the reaction of iron (III) ions with xylenol orange (XO) [41]. The absorbance of the Fe-XO complex was measured at a 560 nm wavelength.

### 2.9. Statistical Analysis

The statistical analysis of the results was performed using GraphPad Prism 7.0 program for MacOS (GraphPad Software, La Jolla, USA). The D'Agostino-Pearson test and Shapiro-Wilk test were used to evaluate the distribution of results. For normal distribution, Student's *t*-test was used and, in case of its absence, the Mann-Whitney *U* test. Correlations between redox biomarkers were assessed using Pearson's correlation coefficient. All the data was presented as the mean, standard deviation (SD), median, minimum, and maximum using tables or charts from individual data points. The analysis of the diagnostic usefulness of redox biomarkers was evaluated by receiver operating characteristic (ROC) analysis. A statistically significant value was *p* ≤ 0.05. The number of patients was determined based on a previous pilot study. The power of the test was assumed to be 0.9.

## 3. Results

### 3.1. Patients

The study did not reveal any significant statistical differences in the clinical characteristics of patients with plaque psoriasis compared to the control. Detailed patient data is presented in Table 1.

### 3.2. Dental Indicators

There were no statistically significant differences in the values of caries intensity indices (DMFT) or indicators describing the condition of marginal periodontium (GI, PBI) in the study group patients compared to healthy controls (Table 2).

### 3.3. Function of Salivary Glands

The secretion of NWS and SWS did not differ significantly in patients with plaque psoriasis compared to the controls. Total protein content was statistically considerably lower in both NWS (*p* = 0.006) and SWS (*p* ≤ 0.001) in plaque psoriasis patients vs. healthy controls. The activity of salivary amylase was significantly lower in NWS and SWS of psoriatic patients vs. healthy subjects (*p* ≤ 0.001 and *p* < 0.001, respectively) (Table 3).

### 3.4. Antioxidative Defence

**Figure 1:**
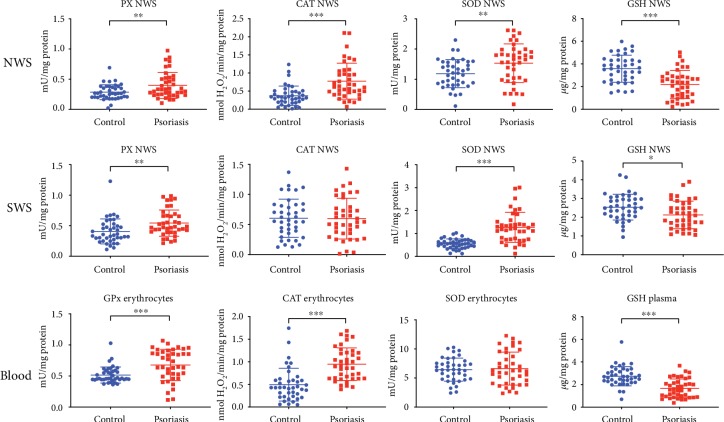
Antioxidant barrier in nonstimulated and stimulated saliva as well as erythrocytes and plasma of psoriasis patients and healthy controls. Abbreviations: CAT: catalase; GPx: glutathione peroxidase; GSH: reduced glutathione; NWS: nonstimulated whole saliva; Px: peroxidase; SOD: superoxide dismutase; SWS: stimulated whole saliva. ^∗^ < 0.05, ^∗∗^ < 0.01, and ^∗∗∗^ < 0.001.

The activity of all antioxidant enzymes in NWS of patients with plaque psoriasis was significantly higher than that in the controls (Px, *p* = 0.0051; CAT, *p* ≤ 0.001; SOD, *p* = 0.004). In SWS of patients with plaque psoriasis, we observed considerably higher activity of Px (*p* = 0.003) and SOD (*p* ≤ 0.001) than that of the control group. In erythrocytes of psoriatic patients, the activity of enzymatic antioxidants GPx and CAT (*p* ≤ 0.001 and *p* ≤ 0.001, respectively) was statistically higher in comparison with that of the control group. We only observed a considerably higher value of TAC (*p* < 0.001) in SWS of patients with plaque psoriasis vs. the controls (Figure 1).

### 3.5. Total Antioxidant Status/Total Oxidant Status and ROS Production

**Figure 2:**
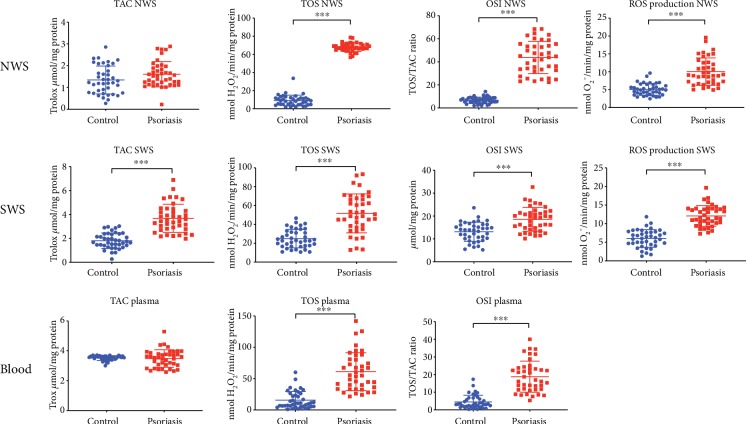
Redox status and ROS production in nonstimulated and stimulated saliva as well as erythrocytes of psoriasis patients and the control group. Abbreviations: NWS: nonstimulated whole saliva; OSI: oxidative stress index; ROS: reactive oxygen species; SWS: stimulated whole saliva; TAC: total antioxidant capacity; TOS: total oxidative status. ^∗^ < 0.05, ^∗∗^ < 0.01, and ^∗∗∗^ < 0.001.

In NWS, SWS, and plasma of patients with plaque psoriasis, we found significantly higher values of TOS and OSI (*p* ≤ 0.001, *p* ≤ 0.001, and *p* ≤ 0.001, respectively) compared to those of the control group. Additionally, in NWS and SWS of psoriatic patients, we observed a faster rate of reactive oxygen species production (*p* < 0.001 and *p* < 0.001, respectively) (Figure 2) compared to that of the control.

### 3.6. Markers of Oxidative Damage to Proteins and Lipids

**Figure 3:**
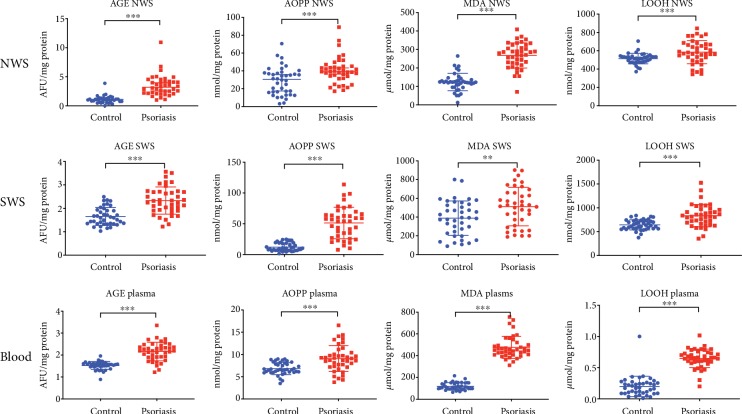
Markers of oxidative damage to proteins and lipids in nonstimulated and stimulated saliva as well as plasma of patients with psoriasis vs. healthy controls. Abbreviations: AGE: advanced glycation end products; AOPP: advanced oxidation protein products; LOOH: total lipid hydroperoxides; MDA: malondialdehyde; NWS: nonstimulated whole saliva; SWS: stimulated whole saliva. ^∗^ < 0.05, ^∗∗^ < 0.01, and ^∗∗∗^ < 0.001.

In NWS, SWS, and plasma of patients with plaque psoriasis, we demonstrated significantly higher concentrations of the protein oxidation markers: AGEs (*p* < 0.001, *p* < 0.001, and *p* < 0.001, respectively) and AOPP (*p* < 0.001, *p* < 0.001, and *p* < 0.001, respectively) vs. healthy controls. The activity of MDA was considerably higher in NWS, SWS, and plasma of psoriatic subjects (*p* < 0.001, *p* = 0.009, and *p* = 0.001, respectively) compared to the control group. The concentration of LOOH was also significantly higher in NWS, SWS, and plasma of plaque psoriasis patients (*p* < 0.001, *p* < 0.001, and *p* < 0.001, respectively) vs. healthy subjects (Figure 3).

### 3.7. Receiver Operating Characteristic (ROC) Analysis

ROC analysis was performed in order to evaluate the diagnostic value of salivary redox biomarkers. The area under the curve (AUC) for AGE concentration in NWS was 0.95. AGE concentration in NWS above 1.69 *μ*mol/mg of protein, at which the specificity and sensitivity was 90%, enables the differentiation of patients with plaque psoriasis vs. healthy patients. The measurement of TOS and OSI concentrations in NWS at 100% specificity and sensitivity allows us to distinguish between healthy and psoriatic patients (Table 4).

The AUC value for ROS production in SWS was 0.96, which means that the rate of ROS production above 8.58 nmol O_2_^−^/min/mg protein at 90% sensitivity and specificity enables the differentiation between patients with plaque psoriasis and healthy subjects. AOPP concentration in SWS, which is a cut-off point and at the same time a value differentiating patients with plaque psoriasis from healthy controls, was 21.37 AFU/mg of protein at 85% specificity and sensitivity (Table 5).

The value of AUC for CAT activity in erythrocytes of patients with plaque psoriasis was 0.85. At the concentration above 63 nmol H_2_O_2_/min/mg protein, with 80% sensitivity and 80% specificity, patients with psoriasis can be clearly distinguished from healthy subjects. MDA concentration in erythrocytes above 1264.00 *μ*mol/mg of protein, at which specificity and sensitivity was 100%, allows us to differentiate between patients with plaque psoriasis and the controls. The measurement of LOOH and MDA concentrations in erythrocytes at 95% and 100% sensitivity and specificity, respectively, enabled the classification of subjects into psoriatic patients and healthy subjects (Table 6).

### 3.8. Correlations

**Figure 4:**
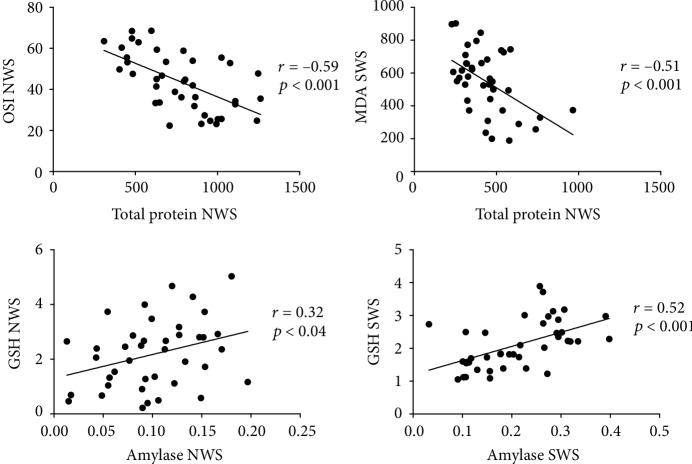
Correlations between selected parameters of oxidative stress and salivary gland function in psoriasis patients. Abbreviations: GSH: reduced glutathione; MDA: malondialdehyde; NWS: nonstimulated whole saliva; OSI: oxidative stress index; SWS: stimulated whole saliva.

**Figure 5:**
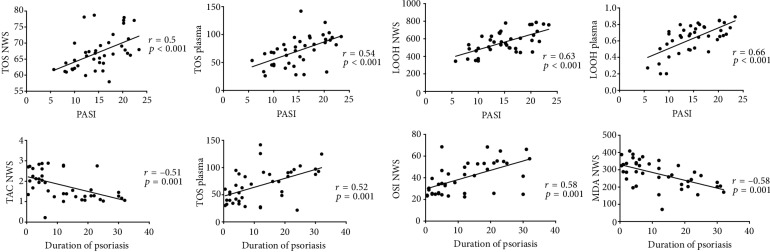
Correlations between selected parameters of oxidative stress and clinical status in psoriasis patients. Abbreviations: LOOH: total lipid hydroperoxides; NWS: nonstimulated whole saliva; OSI: oxidative stress index; PASI: psoriasis area and severity index; TAC: total antioxidant capacity; TOS: total oxidative status.

The results of statistically significant correlations are presented in Figures 4 and 5. In the group of patients with psoriasis, the concentration of OSI in NWS and MDA in SWS correlated negatively with the concentration of total protein. GSH concentration in NWS and SWS correlated positively with salivary amylase activity in psoriatic patients. Moreover, TOS concentration in NWS and plasma as well as the level of LOOH in NWS and plasma correlated positively with PASI. Similar correlations were observed for TAS and OSI in NWS as well as TOS in plasma in relation to the duration of the disease. MDA concentration in NWS also correlated negatively with the duration of psoriasis.

## 4. Discussion

Our study was the first to evaluate the diagnostic utility of salivary biomarkers of oxidative stress in saliva of patients with plaque psoriasis. We also demonstrated that plaque psoriasis disturbs enzymatic antioxidant systems in saliva and leads to oxidative damage to salivary proteins and lipids. We observed that intensification of oxidative modifications of proteins and lipids in saliva correlated with the disease duration.

Oxidative stress can be assessed by means of various biomarkers, including the concentration/activity of nonenzymatic and enzymatic antioxidants, or concentrations of the products of oxidative modifications to cellular components [42]. Extremely useful OS markers are also TAC, TOS, and OSI [9]. TAC is the sum of both enzymatic and nonenzymatic antioxidants, TOS enables the evaluation of the content of all oxidants in a sample, and OSI indicates the relationship between antioxidant mechanisms and oxidant concentrations [43].

Growing evidence supports the hypothesis that increased ROS production and disturbed redox status are implicated in the onset and progress of psoriasis. Typical features of skin lesions in psoriasis include intensified angiogenesis and occurrence of a mixed epidermo-dermal mononuclear inflammatory infiltrate rich in T-cells as well as activated macrophages, mast cells, and neutrophils. In stimulated phagocytic cells, respiratory processes are activated and large amounts of reactive oxygen and nitrogen species are formed. The results of our study demonstrated increased erythrocyte activity of glutathione and catalase peroxidase, reduced glutathione concentration, and a boost in oxidative modifications of proteins and lipids in the plasma of patients with plaque psoriasis compared to the controls, which confirms the previously documented redox balance disturbances and the existence of general oxidative stress in the course of plaque psoriasis [12, 13]. According to the available evidence, lipid peroxidation is one of the factors dysregulating cell proliferation and apoptosis through the mechanism of phospholipase A_2_ activation, production of arachidonic acid derivatives, deactivation of adenylyl cyclase, and activation of guanylyl cyclase [44]. In light of these reports as well as based on the observed correlation between LOOH concentration and PASI, we can conclude that OS is a key factor in the progression and worsening of the plaque psoriasis course.

It is worth noting that there have been no studies evaluating redox balance in the oral cavity of psoriatic patients.

It is believed that about 60% of NWS is produced by submandibular glands, while the parotid, sublingual, and minor salivary glands produce about 25%, 7–8%, and 7–8% of whole saliva, respectively [45]. Stimulation increases saliva secretion by 10–15%, but only in the parotid glands, so it is believed that SWS is a product of parotid glands. Therefore, any changes in the content or secretion of stimulated saliva reflect parotid gland dysfunction. Similarly, changes in the secretion and content of nonstimulated saliva are related to the altered function of submandibular glands [22].

The only antioxidant enzyme of salivary origin is Px. Its significant increase in the secretion of both salivary glands proves that salivary antioxidant systems are capable of responding to the growth of free oxygen radicals in order to enhance cell resistance to OS. CAT and Px participate in the inactivation of hydrogen peroxide formed in the dismutation reaction catalysed by SOD, with Px having a higher affinity to peroxide than CAT [46]. Therefore, it appears that no changes or a slight decrease in CAT activity in SWS may indicate low concentration of hydrogen peroxide at which the enzyme is not activated, and the function of hydrogen peroxide scavenger is served entirely by Px. Indeed, according to Drewa et al. [18], a 20% decrease in CAT activity in the blood of psoriasis patients compared to the controls explains the high concentration of peroxide ions that inhibit catalase activity. On the other hand, such a situation results from oxidative modifications of the polypeptide chain of this enzyme, which—for unknown reasons—is more sensitive to ROS than the one originating from submandibular glands.

The available evidence indicates that thiol groups of glutathione are in balance with thiol groups of proteins, which enables the maintenance of functional activity of proteins as well as a reduced status of protein thiol groups [47]. With 40% decrease in glutathione concentration in NWS and 16.7% in SWS, a positive correlation between glutathione concentration and salivary amylase activity in both salivary glands is therefore not surprising and neither is 58% decrease in activity of this enzyme in NWS and 36.7% in SWS.

Despite a significant 104% increase in TAC in SWS and an insignificant 19.35% increase of this parameter in NWS, the obtained results indicate that the antioxidant defence of salivary glands is insufficient to combat ROS. We observed oxidative stress, which was evidenced by an enhanced level of oxidatively modified cellular constituents and increased OSI [48–50]. ROS can damage all classes of molecular cell components. It was evidenced that a single oxidative damage marker is of limited diagnostic and prognostic value. It is also insufficient to assess the extent of oxidative damage to a given organ [22]. Therefore, in our experiment, we determined the concentrations of MDA, LOOH, AGE, and AOPP to measure oxidative stress.

Our results showed that both nonstimulated and stimulated saliva of psoriatic patients was characterized by increased concentrations of AGE (↑217.3% and ↑41.2%, respectively), AOPP (↑40.8% and ↑323.4%, respectively), MDA (↑116.5% and ↑131.63%, respectively), and LOOH (↑13.8% and ↑30%, respectively). Analysing the above results, we confirmed that a slightly higher percentage of increase in the concentrations of the majority of analytes was observed in the stimulated vs. nonstimulated saliva of psoriatic patients. We also proved increased oxidative damage to parotid vs. submandibular glands in psoriatic patients. However, the intensity of oxidative stress determined by the OSI showed that submandibular (↑550.9%), and not parotid (↑41.5%), glands were more exposed to oxidative attacks of oxygen free radicals generated in the course of psoriasis. Higher intensity of oxidative stress, weaker antioxidative defence (TAC), and higher TOS concentration (submandibular glands 640.55% vs. parotid glands 107.28) in submandibular glands compared to parotid glands can be explained by the fact that parotid glands are highly aerobic organs with a highly efficient mitochondrial antioxidant system, almost as efficient as that of liver mitochondria, capable of inactivating most of the emerging ROS [51, 52]. Moreover, the mechanisms of antioxidative defence in the submandibular glands of patients with plaque psoriasis deteriorate/exhaust along with the duration of the disease (negative correlation between psoriasis duration and TAC). Analysing the presented results, a negative correlation between the disease duration and MDA level could be surprising. However, the decrease in MDA concentration with the duration of the disease can be explained in that aldehydes are capable of reacting with thiol groups of proteins as well as amino groups of proteins, lipids, amino sugars, and nitrogen bases, which leads to the formation of Schiff bases [53]. In addition, cellular mechanisms involved in protecting against the adverse effect of lipid peroxidation may increase with the duration of the disease and can be an adaptive mechanism of the body; e.g., reactive aldehydes are detoxified by glutathione S-transferase [54]. Therefore, both Schiff bases and lipid detoxification products are not detected by reaction with thiobarbituric acid.

The ability of salivary glands of psoriasis patients to secrete saliva at rest and in response to a stimulus was normal. However, unlike saliva secretion, mechanisms responsible for the synthesis/secretion of proteins seemed more sensitive to the effects induced by psoriasis. Protein concentration was significantly decreased in NWS and SWS, being more reduced in SWS (55%) than in NWS (27%). These results as well as the negative correlation between the concentration of protein in the parotid glands and MDA, and the negative correlation between the OSI and the concentration of protein in the parotid glands indicate that the mechanisms associated with protein synthesis/section appear highly sensitive to ROS, as opposed to saliva secretion. It may be assumed that ROS-dependent activation of metalloproteinases, followed by extracellular matrix reconstruction and acinar degeneration, disturbs the function and communication of neural residues and the residual secretory units, which disturbs the release of neurotransmitters and inhibits the response of follicular cells [22]. It was demonstrated that increased catabolism of the extracellular matrix of salivary glands, resulting from increased activity of metalloproteinases, entails the reduction of the active secretory surface of salivary glands as well as the number of nerve fibres, which prevents interaction between a neurotransmitter and receptors on the surface of follicular cells, leading to the reduction of protein concentration [55, 56].

Recently, the use of redox salivary biomarkers in the diagnosis of systemic diseases has been increasingly indicated [9, 22, 25]. The fact is not surprising as the concentration of numerous substances in saliva correlates with their content in the blood serum, and saliva is a noninvasive, noninfectious, and relatively stable laboratory material. In our study, we also assessed the diagnostic usefulness of salivary oxidative stress biomarkers by analysing the area under the curve (AUC). Although many of the examined parameters clearly differentiated psoriatic patients from healthy controls, the evaluation of TOS and OSI in nonstimulated saliva (AUC = 1.0, sensitivity = 100%, and specificity = 100%) deserves special attention. The diagnostic value of TOS and OSI is also emphasized by the positive correlation between the disease duration as well as PASI and the content of TOS and OSI in NWS and plasma. So far, no laboratory biomarkers have been found to determine the severity of psoriasis; therefore, the use of salivary redox diagnostics may be hope for clinicians. Since for our experiment we qualified patients with similar severity of psoriasis (PASI 14.38 ± 4.84), we would like to point out the need for further studies assessing the diagnostic usefulness of salivary redox biomarkers in differentiating the intensity of the disease.

The limitation of the paper is the age range of the study groups. However, we want to emphasize that there were only 4 patients over 60 years of age, as well as that the results obtained for these patients did not differ significantly as compared to those for other patients from the study group. We would also like to point out that the control group is selected by age and gender in relation to the study group, which at least eliminates the impact of age on the assessed redox biomarkers.

## 5. Conclusions

We observed that the course of plaque psoriasis is accompanied by redox imbalances with the prevalence of oxidation reactions. Both parotid and submandibular glands of psoriatic patients had impaired ability to maintain normal redox balance, resulting in an elevated concentration of oxidized biomolecules vs. that of the control. However, redox equilibrium in submandibular glands was more vulnerable as well as the antioxidant capacity of submandibular glands decreases with the duration of the disease. It should be underlined that mechanisms involved in the synthesis/secretion of proteins were disrupted in both types of salivary glands of psoriatic patients, being more reduced in SWS than in NWS. We claim that salivary TOS and OSI may be potential diagnostic biomarkers of plaque psoriasis.

## Figures and Tables

**Table 1:** Clinical characteristics of psoriasis patients and the control group.

Patient characteristics	Control *n* = 40	Psoriasis *n* = 40
Sex		
Male *n* (%)	27 (67.50)	27 (67.50)
Female *n* (%)	13 (32.50)	13 (32.50)
Age	45.44 ± 19.28	45.55 ± 20.18
Height (cm)	169.23 ± 11.28	174.40 ± 10.78
Weight (kg)	80.58 ± 13.71	82.02 ± 14.75
Duration of psoriasis	—	12.71 ± 9.803
PASI	—	14.38 ± 4.84
Psoriasis in the family		
<1 *n* (%)	—	9 (22.50)
≥1 *n* (%)	—	31 (77.50)
WBC (10^3^/*μ*L)	5.83 ± 1.97	6.74 ± 2.05
RBC (10^6^/*μ*L)	4.35 ± 0.46	4.59 ± 0.56
HCT (%)	42.28 ± 4.03	41.08 ± 3.57
PLT (10^3^/*μ*L)	235.42 ± 51.44	227.50 ± 62.62
CRP (mg/L)	4.98 ± 9.26	5.25 ± 8.58
TG (mg/dL)	127.81 ± 80.27	138.70 ± 72.55
CHOL (mg/dL)	151.92 ± 36.24	176.60 ± 29.70
Glc (mg/dL)	76.38 ± 10.53	85.38 ± 9.73
ALT (U/L)	23.67 ± 19.28	25.95 ± 21.01
AST (U/L)	24.85 ± 17.51	25.67 ± 18.25
Blood pressure		
Systolic (mmHg)	125.86 ± 19.20	130.70 ± 17.60
Diastolic (mmHg)	78.61 ± 13.72	83.14 ± 10.40

Abbreviations: ALT: alanine transferase; AST: aspartate transaminase; CHOL: cholesterol; CRP: C-reactive protein; Glc: D-glucose; HCT: haematocrit; *n*: number of patients; PASI: psoriasis area and severity index; PLT: platelets; RBC: red blood cells; TG: triglyceride; WBC: white blood cells.

**Table 2:** Dental characteristics of psoriasis patients and control subjects.

Patient characteristics	Control		Psoriasis	
	*n* = 40		*n* = 40	
	Mean ± SD	Median (min–max)	Mean ± SD	Median (min–max)
DMFT	17.68 ± 5.62	15.00 (4.00–28.00)	18.23 ± 6.81	16.00 (6.00–28.00)
PBI	0.61 ± 0.58	0.48 (0–2.12)	0.53 ± 0.55	0.41 (0–2.02)
GI	0.31 ± 0.31	0.23 (0–1.25)	0.32 ± 0.36	0.21 (0–1.56)

Abbreviations: DMFT: decayed, missing, filled teeth index; GI: gingival index; *n*: number of patients; PBI: papilla bleeding index. *p* < 0.05 vs. the control group.

**Table 3:** Salivary gland function in psoriasis patients and control subjects.

Patient characteristics	Control		Psoriasis	
	*n* = 40		*n* = 40	
	Mean ± SD	Median (min–max)	Mean ± SD	Median (min–max)
NWS flow rate (mL/min)	0.31 ± 0.17	0.28 (0.10–0.89)	0.28 ± 0.14	0.24 (0.10–0.63)
SWS flow rate (mL/min)	0.95 ± 0.61	0.99 (0.23–3.5)	0.88 ± 0.51	0.76 (0.2–2.4)
Total protein in NWS (*μ*g/mL)	1184.00 ± 536.60	1190.00 (180.60–2127.00)	865.50 ± 300.80	857.60 (309.50–1280.00)^∗^
Total protein in SWS (*μ*g/mL)	977.80 ± 402.20	917.10 (478.00–2002.00)	439.50 ± 151.10	439.20 (227.90–965.40)^∗^
Amylase in NWS (*μ*mol/mg protein)	0.24 ± 0.07	0.23 (0.03–0.39)	0.10 ± 0.05	0.10 (0.01–0.20)^∗^
Amylase in SWS (*μ*mol/mg protein)	0.29 ± 0.14	0.30 (0.03–0.59)	0.19 ± 0.09	0.16 (0.01–0.40)^∗^

Abbreviations: *n*: number of patients; NWS: nonstimulated whole saliva; SWS: stimulated whole saliva. ^∗^*p* < 0.05 vs. the control group.

**Table 4:** ROC analysis of antioxidant defence, total antioxidant/oxidant status, and markers of oxidative damage to proteins and lipids in nonstimulated saliva of psoriasis patients compared to the controls.

Parameter	NWS					
	AUC	95% confidence interval	*p* value	Cut-off	Sensitivity	Specificity
Px (mU/mg protein)	0.65	0.53 to 0.7713	**0.0204**	>0.29	57.50	57.50
CAT (nmol H_2_O_2_/min/mg protein)	0.79	0.6861 to 0.8864	**<0.0001**	>0.47	72.50	72.50
SOD (mU/mg protein)	0.68	0.5634 to 0.8029	**0.0048**	>1.39	62.50	62.50
GSH (*μ*g/mg protein)	0.78	0.6839 to 0.8823	**<0.0001**	<2.81	70.00	70.00
TAC (Trolox *μ*mol/mg protein)	0.62	0.4949 to 0.7451	0.0647	>1.37	57.50	57.50
TOS (nmol H_2_O_2_/min/mg protein)	1.00	1 to 1	**<0.0001**	>45.26	100.00	100.00
OSI (TOS/TAC ratio)	1.00	1 to 1	**<0.0001**	>18.30	100.00	100.00
ROS production (nmol O_2_^−^/min/mg protein)	0.91	0.8501 to 0.9699	**<0.0001**	>6.55	80.00	80.00
AGE (AFU/mg protein)	0.95	0.902 to 0.9993	**<0.0001**	>1.69	90.00	90.00
AOPP (nmol/mg protein)	0.73	0.6174 to 0.8389	**0.0004**	>36.07	67.50	67.50
MDA (*μ*mol/mg protein)	0.95	0.9028 to 1.002	**<0.0001**	>187.80	87.50	87.50
LOOH (nmol/mg protein)	0.72	0.5933 to 0.8367	**0.0009**	>534.00	67.50	67.50

Abbreviations: AGE: advanced glycation end products; AOPP: advanced oxidation protein products; AUC: area under curve; CAT: catalase; GSH: reduced glutathione; LOOH: total lipid hydroperoxides; MDA: malondialdehyde; NWS: nonstimulated whole saliva; OSI: oxidative stress index; ROS: reactive oxygen species; Px: peroxidase; SOD: superoxide dismutase; TAC: total antioxidant capacity; TOS: total oxidative status.

**Table 5:** ROC analysis of antioxidant defence, total antioxidant/oxidant status, and markers of oxidative damage to proteins and lipids in stimulated saliva of psoriasis patients compared to the controls.

Parameter	SWS					
	AUC	95% confidence interval	*p* value	Cut-off	Sensitivity	Specificity
Px (mU/mg protein)	0.69	0.5784 to 0.8091	**0.0029**	>0.44	62.50	62.50
CAT (nmol H_2_O_2_/min/mg protein)	0.51	0.3854 to 0.6408	0.8399	<0.55	52.50	52.50
SOD (mU/mg protein)	0.87	0.7902 to 0.9523	**<0.0001**	>0.73	80.00	80.00
GSH (*μ*g/mg protein)	0.67	0.5494 to 0.7881	**0.0094**	<2.32	62.50	62.50
TAC (Trolox *μ*mol/mg protein)	0.94	0.8915 to 0.9848	**<0.0001**	>2.43	82.50	82.50
TOS (nmol H_2_O_2_/min/mg protein)	0.88	0.7955 to 0.957	**<0.0001**	>34.97	80.00	80.00
OSI (TOS/TAC ratio)	0.78	0.6823 to 0.8802	**<0.0001**	>15.38	70.00	70.00
ROS production (nmol O_2_^−^/min/mg protein)	0.96	0.9215 to 0.9973	**<0.0001**	>8.58	90.00	90.00
AGE (AFU/mg protein)	0.83	0.7444 to 0.9206	**<0.0001**	>1.92	75.00	75.00
AOPP (nmol/mg protein)	0.95	0.8996 to 0.9941	**<0.0001**	>21.37	85.00	85.00
MDA (*μ*mol/mg protein)	0.67	0.5499 to 0.7863	**0.0096**	>461.50	62.50	62.50
LOOH (nmol/mg protein)	0.79	0.6829 to 0.8871	**<0.0001**	>714.70	70.00	70.00

Abbreviations: AGE: advanced glycation end products; AOPP: advanced oxidation protein products; AUC: area under curve; CAT: catalase; GSH: reduced glutathione; LOOH: total lipid hydroperoxides; MDA: malondialdehyde; OSI: oxidative stress index; ROS: reactive oxygen species; SWS: stimulated whole saliva; Px: peroxidase; SOD: superoxide dismutase; TAC: total antioxidant capacity; TOS: total oxidative status.

**Table 6:** ROC analysis of antioxidant defence, total antioxidant/oxidant status, and markers of oxidative damage to proteins and lipids in erythrocytes and plasma of psoriasis patients compared to the controls.

Parameter						
	AUC	95% confidence interval	*p* value	Cut-off	Sensitivity	Specificity
Erythrocytes						
GPx (mU/mg protein)	0.73	0.609 to 0.8472	**0.0004**	>0.55	65.00	65.00
CAT (nmol H_2_O_2_/min/mg protein)	0.85	0.7564 to 0.9336	**<0.0001**	>0.63	80.00	80.00
SOD (mU/mg protein)	0.51	0.3811 to 0.6414	0.8625	<6.29	57.00	57.00
Plasma						
GSH (*μ*g/mg protein)	0.82	0.7205 to 0.9108	**<0.0001**	<2.21	75.00	75.00
TAC (Trolox *μ*mol/mg protein)	0.5	0.3618 to 0.6457	0.954	<3.56	52.50	52.50
OSI (TOS/TAC ratio)	0.96	0.916 to 0.9953	**<0.0001**	>8.81	85.00	85.00
AGE (AFU/mg protein)	0.92	0.8492 to 0.9895	**<0.0001**	>1.68	87.5	87.5
AOPP (nmol/mg protein)	0.77	0.6554 to 0.8746	**<0.0001**	>7.73	70.00	70.00
MDA (*μ*mol/mg protein)	1.00	1 to 1	**<0.0001**	>264.00	100.00	100.00
LOOH (nmol/mg protein)	0.96	0.9091 to 1.013	**<0.0001**	>0.36	95.00	95.00

Abbreviations: AGE: advanced glycation end products; AOPP: advanced oxidation protein products; AUC: area under curve; CAT: catalase; GSH: reduced glutathione; GPx: glutathione peroxidase; LOOH: total lipid hydroperoxides; MDA: malondialdehyde; OSI: oxidative stress index; SOD: superoxide dismutase; TAC: total antioxidant capacity.

## Data Availability

The data used to support the findings of this study are available from the corresponding author upon request.
